# Biochemical characteristics, genetic variants and treatment outcomes of 55 Chinese cases with neonatal intrahepatic cholestasis caused by citrin deficiency

**DOI:** 10.3389/fped.2024.1293356

**Published:** 2025-01-13

**Authors:** Juan Li, Jintao Duan, Shuli He, Ying Li, Meifen Wang, Chengjun Deng

**Affiliations:** ^1^ Department of Gastroenterology, Kunming Children’s Hospital, Kunming, China; ^2^ Department of Infectious Diseases, Kunming Children’s Hospital, Kunming, China

**Keywords:** NICCD, nutritional assessment, biochemical characteristics, SLC25A13, LF/MCT formula

## Abstract

**Background:**

The diagnostic criteria of neonatal intrahepatic cholestasis caused by citrin deficiency (NICCD) have not been established due to non-specific clinical manifestations, and our understanding on the treatment outcome is still limited. We aim to investigate the biochemical characteristics, genetic variants, and treatment outcome of NICCD patients.

**Methods:**

We compared the nutritional status and biochemical characteristics of 55 NICCD infants and 27 idiopathic neonatal cholestasis (INC) infants. *SLC25A13* gene variant analysis was performed for definitive diagnosis of NICCD. NICCD infants received 12 months of lactose-free and/or medium-chain triglyceride-enriched (LF/MCT) formula treatment. The treatment efficacy was evaluated by comparing the outcome of NICCD with the 24 healthy infants that were selected as normal controls. All NICCD patients were followed up until death or at least 1 year of age.

**Results:**

Compared to INC group, significant increase was found in levels of total bilirubin, indirect bilirubin, total bile acid, gamma-glutamyl transpeptidase, alkaline phosphatase, prothrombin time, thrombin time, international normalized ratio, alpha-fetoprotein (AFP), Vitamin D, and Vitamin E of NICCD group, while alanine aminotransferase, albumin, fibrinogen, glucose, and Vitamin A levels showed significant decrease in the NICCD group (*P* < 0.05). There were 7 novel variants among 19 *SLC25A13* variant types. No significant differences were found between NICCD patients treated for 12 months and normal controls. In long term follow-up, 2 cases developed FTTDCD, 8 cases had special dietary habits, and 1 case died from cirrhosis.

**Conclusions:**

NICCD showed more severe impairments in liver, coagulation, and metabolic function than INC. Significantly increased AFP levels could provide reference for the differential diagnosis of NICCD. The newly discovered variants may be meaningful for the individualized treatment of NICCD patients. LF/MCT formula was recommended for NICCD patients.

## 1 Introduction

Neonatal cholestasis is a common disease in pediatrics with biliary atresia, infection, and inherited metabolic diseases as common causes (1). Neonatal intrahepatic cholestasis caused by citrin deficiency (NICCD, OMIM 605814) is an autosomal recessive disorder resulting in neonatal cholestasis, which is caused by dysfunction of citrin, a liver-type aspartate/glutamate carrier protein located within mitochondrial inner membrane (2). To date, NICCD cases are mainly distributed in East Asian countries such as Japan, China, Korea and Vietnam (3–8). For instance, in a recent selective screening for in inborn errors of metabolism in mainland China, Song et al. revealed that the positive rate of NICCD ranked the second highest just behind methylmalonic aciduria in high-risk Chinese population (9).

The clinical manifestations of NICCD (e.g., jaundice, long-term cholestasis, failure to thrive, liver dysfunction, and metabolic abnormalities including citrullinemia, hyperammonemia, hypoproteinemia, galactosemia, and hypoglycemia) are usually atypical (2, 3, 10, 11). Additionally, its diagnostic criteria have not yet been established due to the complicated heterogeneity and the diversity of gene variants. To date, *SLC25A13* locating on chromosome 7q21.3 has been well acknowledged as a pathologic gene for NICCD (12). It contributes to the timely diagnosis in clinical practice, but there is a long way for the establishing of diagnostic criteria for the NICCD due to a lack of more reliable biochemical and genetic information for it.

Upon diagnosis of NICCD, patients will be timely treated with a dietary management of lactose-free and/or medium-chain triglyceride-enriched (LF/MCT) formula (3). Most patients showed remarkable response to the treatment within the first year, but unfortunately, some may present failure to thrive and dyslipidemia caused by citrin deficiency (FTTDCD) with growth retardation and dyslipidemia as the main manifestations (13). Although a few patients show remission of NICCD in a short term, they may develop severe adult-onset type Ⅱ citrullinemia (CTLN2) decades later (14). Therefore, more attention should be paid to the treatment outcomes of NICCD with different genetic variant types, with an aim to develop individualized treatment strategies. In the present study, we focused on the biochemical characteristics, genetic variants and outcomes between infants with NICCD.

## 2 Materials and methods

### 2.1 Subjects

Infants with NICCD diagnosed by clinical manifestations and variant analysis of the *SLC25A13* gene were collected from our hospital from February 2016 to January 2023. Infants who met the following criteria were included: (i) onset age <1 year old; (ii) diagnosed with intrahepatic cholestasis; (iii) carrying homozygous or heterozygous variant of *SLC25A13* gene; (iv) with complete clinical data; and (v) follow-up on time after discharge. We excluded infants whose parents were unwilling to participate in the study and those with incomplete clinical data.

Infants with idiopathic neonatal cholestasis (INC) who visited our department during the same period were included in the INC group. Inclusion criteria were: (i) infants with an onset age <1 year old; (ii) infants met the diagnostic criteria recommended by the ESPGHAN: a direct bilirubin (DB) level exceeding 20% of the total bilirubin (TB), or a DB level greater than 1 mg/dl (17 μmol/L); and iii) infants who had complete clinical data. Infants with the following conditions were excluded: (i) those with cytomegalovirus infection, hepatitis virus infection, biliary tract inflammation, and enterovirus infection; (ii) with biliary tract diseases such as biliary atresia, biliary stricture, choledochal cyst, and tumor; and (iii) carrying variants of *SLC25A13* gene or/and other genes related to genetic liver diseases.

The studies involving humans were approved by the ethical committee of Kunming Children's Hospital (No. 2022-03-078-K01). The studies were conducted in accordance with the local legislation and institutional requirements. Written informed consent for participation in this study was provided by the participants’ legal guardians/next of kin.

### 2.2 General and biochemical data collection

We collected the general information at enrollment of each infant in the NICCD and INC groups, including gender, birth weight, onset age, age at presentation, weight and height at admission, as well as weight for age z-score (WAZ), height for age z-score (HAZ), and weight for height z-score (WHZ) calculated by the WHO Anthm software. Besides, the biochemical characteristics at enrollment were investigated between the NICCD and INC groups, including levels of hemoglobin (HGB), liver function indices [alanine aminotransferase (ALT), aspartate aminotransferase (AST), TB, DB, indirect bilirubin (IB), total bile acid (TBA), gamma-glutamyl transpeptidase (GGT), alkaline phosphatase (AKP), albumin (ALB)], coagulation parameters [prothrombin time (PT), activated partial thromboplastin time (APTT), fibrinogen (FIB), thrombin time (TT), international normalized ratio (INR)], metabolic indices [glucose (Glu), total cholesterol (TCH), triglyceride (TG), blood ammonia (AMON), alpha-fetoprotein (AFP), lactic acid (Lac)], as well as vitamins (VA, VD, VE).

### 2.3 Nutritional assessment criteria

Low birth weight (LBW) was defined as weight at birth of less than 2.5 kg (15). WAZ values of −2 to −1 indicated a mild malnutrition, −3 to −2 a moderate one and lower than −3 a severe malnutrition. Growth retardation was defined as HAZ values of less than −2. WHZ values of less than −2 were considered emaciation.

VA and VE levels were assessed according to previous criteria (16): A concentration of <0.70 μmol/L, 0.70–1.05 μmol/L and >1.05 μmol/L for VA was designated as deficiency, marginal deficiency, and normal, respectively. For VE, a concentration of <12.0 μmol/L, 12.0–16.7 μmol/L and 16.8–33.5 μmol/L was designated as deficiency, decrease and normal, respectively. The classification criteria for VD levels were as follows (17): normal, 75.1–250 nmol/L; 50.1–75 nmol/L, insufficiency; 25.1–50 nmol/L, mild deficiency; 12.5–25 nmol/L, moderate deficiency; <12.5 nmol/L, severe deficiency.

### 2.4 Next-generation sequencing (NGS)

Peripheral blood (4 ml) was collected from the infants and their family members using ethylenediamine tetraacetic acid (EDTA) anticoagulant tubes. Genomic DNA was extracted using Qiagen FlexiGene DNA Kit (51206, Qiagen, Germany). Quality control for genomic DNA was performed to ensure that at least 1.5 μg genomic DNA of the proband was used for library construction.

NGS was performed with an Illumina NovaSeq 6,000 sequencer (Illumina, USA) by Kangxu Medical Laboratory (Beijing, China) to preliminarily identify suspected variants. The suspected variants were retrieved from dbSNP, 1,000 Genomes, gnom AD, ESP6500, ExAC, Human Gene Mutation Database, as well as domestic and foreign literatures. Pathogenicity of the novel variants was determined according to the *standards and guidelines for the interpretation of sequence variants* proposed by the American College of Medical Genetics and Genomics (ACMG) and the Association for Molecular Pathology (AMP).

Sanger sequencing was then conducted to exclude false positive variant sites. Primers were designed and synthesized on PrimerZ (http://genepipe.ngc.sinica.edu.tw/primerz/). Then PCR was performed using Goldstar Taq mix reagent (40526, Kangwei Century Biotechnology, Beijing, China) on ABI Veriti 96-Well Thermal Cycler (Applied Biosystems, USA) to amplify the sequence of mutant genes such as *SLC25A13* in the infants and their parents, followed by the purification of PCR products via 1% agarose gel electrophoresis. Afterwards, sequencing was performed on an ABI PRISM 3,730 genetic analyzer (Applied Biosystems, USA). Variant sites were identified by comparing the DNA sequences to reference sequences in the GenBank database.

### 2.5 Treatment and follow-up

To evaluate the treatment outcome of the NICCD, age-matched healthy infants who visited our clinics during the same period were randomly selected into normal control group. They showed normal liver function with no jaundice. Besides, they did not have serious organic diseases or chronic diseases such as chronic infectious diseases, chronic hepatorenal diseases, rickets, cardiovascular diseases, hematologic diseases, and autoimmune diseases.

NICCD patients were treated for 12 months from the following aspects. Breast milk and ordinary lactose-containing formula were replaced by lactose-free formula enriched with medium-chain triglyceride (MCT). Ursodeoxycholic acid (sodium salt) was used to alleviate cholestasis, and hepatoprotective drugs were given to improve liver function. A low-carbohydrate, high-protein, and high-lipid dietary treatment was introduced after adding complementary foods within 1 year of age, and all the patients had a normal diet after 1 year of age. Vitamins (VA, VD, VE) were supplemented. Patients were followed up for biochemical changes, dietary preferences, as well as growth and development after discharge. Finally, body weight, WAZ, liver function indices, trace element levels and vitamin levels of the infants in the NICCD group and normal control group were collected to evaluate the treatment outcome.

### 2.6 Statistical analysis

Statistical analysis was performed using SPSS 26.0 software (IBM; Armonk, NY, USA). Continuous variable with a normal distribution were presented as the means ± standard deviations and were analyzed using the student's *t* test. The variables not normally distributed were described as the medians (interquartile ranges) and were compared utilizing Mann-Whitney U test (*z* test). Categorical variables were presented as frequencies and percentages (%), and Chi-square test was used for comparison. All analyses were two-sided, and a *P*-value of <0.05 was considered statistically significant.

## 3 Results

### 3.1 Epidemiological characteristics of the NICCD and INC groups

Fifty-five patients (male: 37; female:18) were included in NICCD group, with a male to female ratio of 2.1:1. The onset age ranged from 1 day to 64 days, of which 31 (56.4%), 15 (27.3%) and 9 (16.4%) cases had an onset age of less than 7 days, 7–30 days, and more than 30 days, respectively. The age at presentation ranged from 1 month to 16 months. Twenty-seven infants were included INC group had a male to female ratio of 1.25:1 (male:15; female: 12). The onset age ranged from 1 day to 30 days, with 22 (81.5%) cases of less than 7 days and 5 (18.5%) cases of 7–30 days. The age at presentation ranged from 1 month and 10 days to 3 years and 8 months. The onset age was significantly different between NICCD and INC groups (*P* < 0.05). No significant difference was found in gender and age at presentation between NICCD and INC groups (*P* > 0.05) (Table 1).

**Table 1:** Epidemiological characteristics and nutritional assessment of the NICCD and INC groups.

Index	NICCD group	INC group	*t* test	Chi-square test	*z* test	*P* value
	(*N* = 55) [n (%), Mean ± SD, or M (Q_1_, Q_3_)]	(*N* = 27) [n (%), Mean ± SD, or M (Q_1_, Q_3_)]				
Epidemiological characteristics						
Male	37 (67.3%)	15 (55.6%)		1.072		0.301
Onset age (d)						
< 7	31 (56.4%)	22 (81.5%)		6.755		0.034
7–30	15 (27.3%)	5 (18.5%)				
> 30	9 (16.4%)	0				
Age at presentation (d)	65.0 (46.0, 90.0)	126 (71, 312)			−0.291	0.771
Nutritional assessment						
Birth weight (kg)	2.95 ± 0.29	2.96 ± 0.59	−0.151			0.881
WAZ at birth	−0.83 ± 0.65	−0.81 ± 1.41	−0.118			0.906
Weight at admission (kg)	4.40 (4.00, 5.20)	4.50 (4.00, 5.40)			−0.143	0.886
WAZ	−1.54 ± 0.82	−1.39 ± 1.02	−0.747			0.457
Height (cm)	55.00 (54.00, 57.00)	56.00 (53.00, 58.00)			−0.040	0.968
HAZ	−1.53 ± 1.13	−1.36 ± 1.28	−0.617			0.539
WHZ	−0.12 (−0.96, 0.39)	−0.12 (−1.27, 0.39)			−0.212	0.832
VA (μmol/L)	0.58 (0.43, 0.80)	1.17 (0.81, 1.71)			−3.833	< 0.001
VD (nmol/L)	54.23 (43.49, 92.15)	41.58 (26.39, 64.88)			−2.520	0.012
VE (μmol/L)	17.72 (15.63, 21.25)	14.70 (13.40, 19.86)			−2.063	0.039

NICCD, neonatal intrahepatic cholestasis caused by citrin deficiency. INC, idiopathic neonatal cholestasis. Mean ± SD denotes mean ± standard deviation; M (Q_1_, Q_3_) means median (the first quartile, the third quartile). WAZ, weight for age z-score; HAZ, height for age z-score; WHZ, weight for height z-score; VA, vitamin A; VD, vitamin D; VE, vitamin E.

### 3.2 Nutritional assessment of the NICCD and INC groups

**Figure 1:**
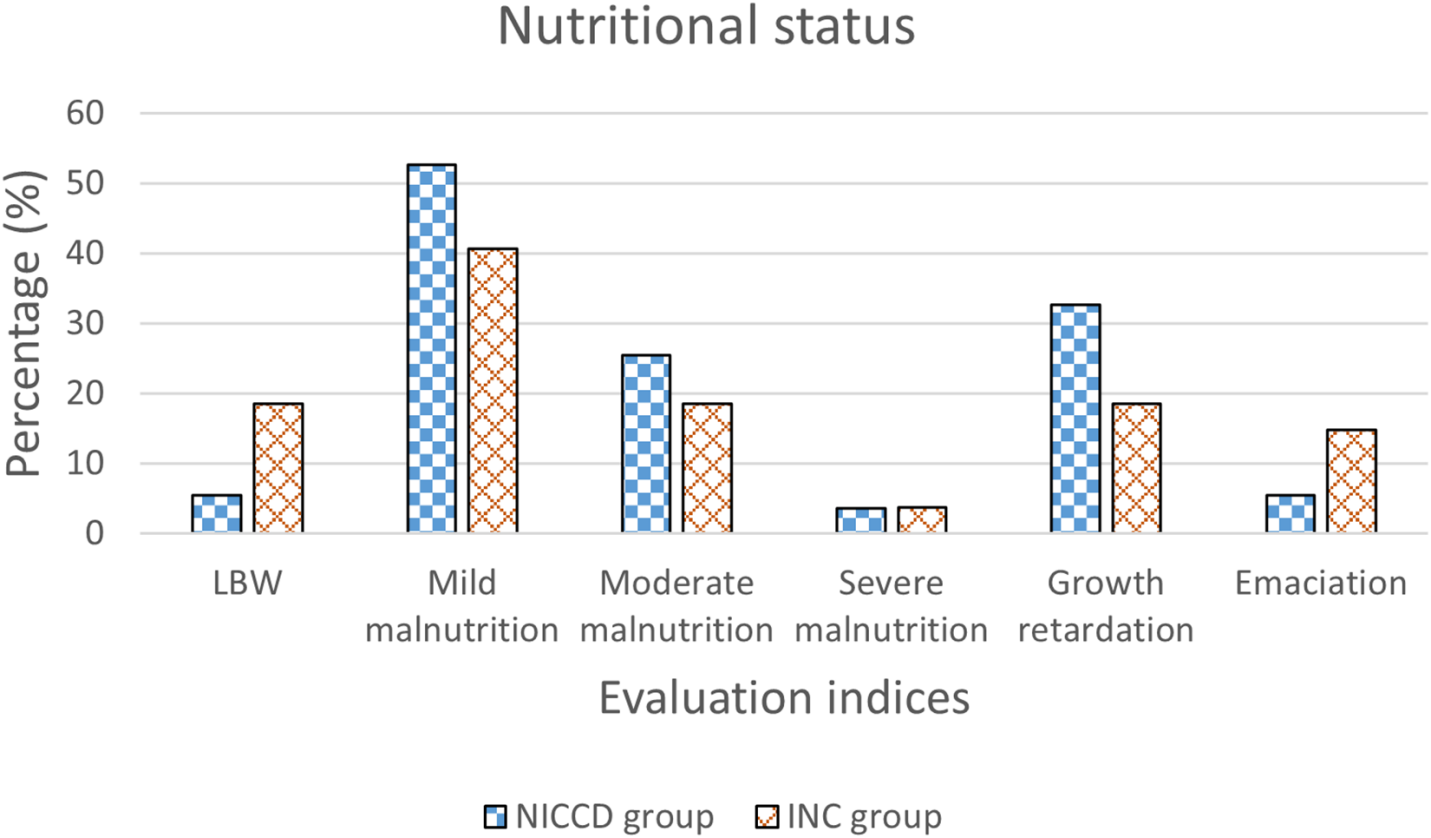
Percentage of infants with LBW, mild malnutrition, moderate malnutrition, severe malnutrition, growth retardation, and emaciation in the NICCD and INC groups. NICCD, neonatal intrahepatic cholestasis caused by citrin deficiency; INC, idiopathic neonatal cholestasis; LBW, low birth weight.

In the NICCD group, there were 3 (5.5%) LBW infants at enrollment. There were 45 (81.8%) infants suffering from malnutrition, including 29 (52.7%) cases of mild malnutrition, 14 (25.5%) cases of moderate malnutrition, and 2 (3.6%) cases of severe malnutrition. Besides, 18 (32.7%) cases showed growth retardation, and 3 (5.5%) cases showed emaciation. In the INC group, 5 (18.5%) LBW infants were found at enrollment. There were 17 (63.0%) infants showing malnutrition, including 11 (40.7%) of mild malnutrition, 5 (18.5%) of moderate malnutrition, and 1 (3.7%) of severe malnutrition. Additionally, the number of patients with growth retardation and emaciation was 5 (18.5%) and 4 (14.8%), respectively (Figure 1).

**Figure 2:**
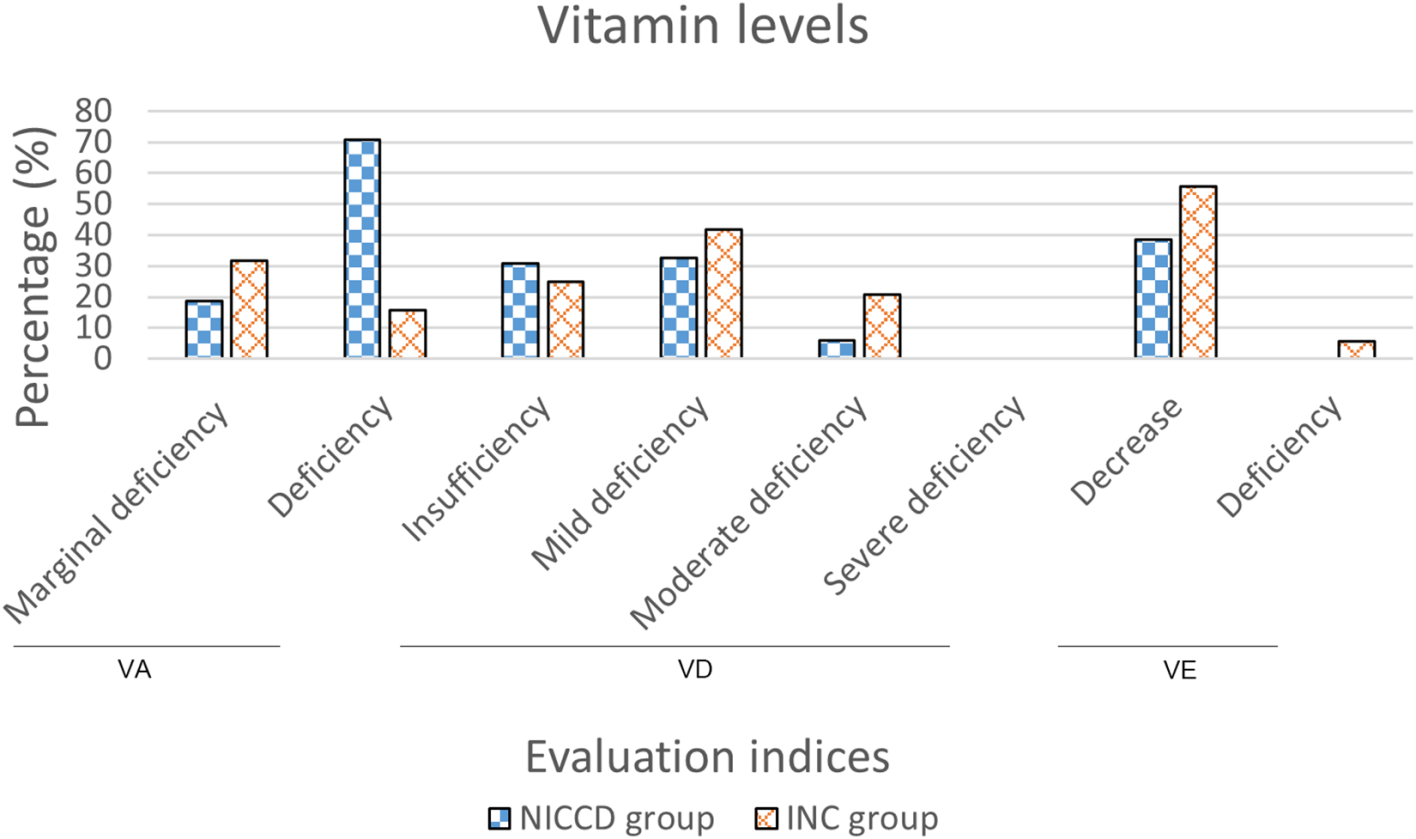
Percentage of infants with abnormal vitamin levels in the NICCD and INC groups. NICCD, neonatal intrahepatic cholestasis caused by citrin deficiency; INC, idiopathic neonatal cholestasis; VA, vitamin A; VD, vitamin D; VE, vitamin E.

We obtained the VA, VD, and VE levels of 48, 52, and 44 infants in the NICCD group, respectively. Forty-three (89.6%) cases had abnormal VA levels, including 9 (18.8%) cases with marginal deficiency and 34 (70.8%) cases with deficiency. There were 36 (69.2%) cases with abnormal VD levels, including 16 (30.8%) cases of insufficiency, 17 (32.7%) cases of mild deficiency, and 3 (5.8%) cases of moderate deficiency. Seventeen (38.6%) cases showed decreased VE levels, and no cases showed deficiency of VE. The VA, VD, and VE levels were obtained from 19, 24, and 18 infants in the INC group, respectively. Nine (47.4%) cases showed abnormal VA levels, including 6 (31.6%) cases of marginal deficiency and 3 (15.8%) cases of deficiency. A total of 21 (87.5%) cases had abnormal VD levels, including 6 (25.0%) cases of insufficiency, 10 (41.7%) cases of mild deficiency, and 5 (20.8%) cases of moderate deficiency. VE levels were abnormal in 11 (61.1%) cases, of which 10 (55.6%) cases were at a decreased level, and 1 (5.6%) case was at a deficient level (Figure 2).

Infants with NICCD showed decrease in VA levels and increase of VD and VE levels than those with INC (*P* < 0.05) (Table 1). No significant differences were found in weight and WAZ at birth, as well as WAZ, WHZ, height, HAZ, and WHZ at admission between NICCD and INC groups.

### 3.3 Biochemical characteristics of the NICCD and INC groups

At diagnosis, all the patients in NICCD and INC groups showed increase in ALT, AST, TB, DB, IB, TBA, GGT, AKP, AFP, and Lac, together with decrease in ALB levels. Several biochemical changes were different between the two groups. PT was excessed, while FIB and Glu were decreased in the NICCD group, however, they were at normal levels in the INC group. Compared to INC group, significant increase was found in TB, IB, TBA, GGT and AKP in the NICCD group (all *P* < 0.05), while significant decrease was shown in ALT and ALB (all *P* < 0.05). NICCD group showed significant changes in coagulation parameters than INC group. Specifically, compared with INIC group, patients in the NICCD group showed significant increase in PT, TT and INR, together with significant decrease in FIB (all *P* < 0.001). In terms of metabolism, compared to INC group, NICCD group showed significant increase in AFP levels and decrease in Glu levels (all *P* < 0.001) (Table 2).

**Table 2:** Comparison of biochemical indices in the NICCD and INC patients at diagnosis.

Index	Reference range	NICCD group	INC group	*t/z* test	*P* value
		(*N* = 55) [Mean ± SD, or M (Q_1_, Q_3_)]	(*N* = 27) [Mean ± SD, or M (Q_1_, Q_3_)]		
HGB (g/L)	–	101.47 ± 13.28	105.33 ± 14.96	−1.186	0.239
ALT (U/L)	7–30	43.00 (29.75, 57.25)	128.00 (60.00, 294.00)	−4.273	< 0.001
AST (U/L)	14–44	116.00 (85.25, 135.75)	126.00 (60.00, 277.00)	−0.927	0.354
TB (μmol/L)	4.3–17.7	151.40 (125.28, 197.70)	102.90 (75.70, 179.10)	−2.199	0.028
DB (μmol/L)	0–3.4	71.70 (55.98, 97.45)	61.15 (38.08, 122.38)	−0.796	0.426
IB (μmol/L)	0–12	78.10 (52.28, 105.03)	33.50 (12.60, 66.70)	−3.647	< 0.001
TBA (μmol/L)	0–10	247.34 ± 75.71	127.12 ± 74.74	6.765	< 0.001
GGT (U/L)	5–19	188.50 (131.00, 231.00)	92.00 (67.00, 179.00)	−3.442	0.001
AKP (U/L)	143–406	907.00 (723.50, 1,327.50)	517.00 (377.00, 659.00)	−5.662	< 0.001
ALB (g/L)	39–54	32.05 ± 4.37	38.16 ± 3.47	−6.322	< 0.001
PT (s)	11.0–14.5	15.70 (12.75, 18.55)	12.60 (11.90, 13.10)	−4.010	< 0.001
APTT (s)	28.0–44.5	43.30 (36.80, 53.65)	39.00 (36.70, 45.30)	−1.791	0.073
FIB (g/L)	2–4	1.09 (0.87, 1.38)	2.42 (2.22, 2.67)	−6.660	< 0.001
TT (s)	14–21	19.60 (21.40, 23.40)	17.60 (16.90, 18.90)	−5.699	< 0.001
INR	0.8–1.5	1.34 (1.05, 1.58)	0.92 (0.89, 1.02)	−5.496	< 0.001
Glu (mmol/L)	3.9–5.8	2.80 ± 1.23	4.12 ± 1.18	−4.621	< 0.001
TCH (mmol/L)	3.12–5.20	4.35 (3.72, 5.62)	3.96 (3.51, 4.94)	−1.386	0.166
TG (mmol/L)	0.8–1.8	0.92 (0.68, 2.37)	1.35 (1.00, 1.93)	−1.119	0.063
AMON(μmol/L)	10–50	34.30 (27.35, 41.35)	32.10 (26.18, 40.35)	−0.151	0.880
AFP (ng/ml)	0–12	60,500.00 (33,737.25, 60,500.00)	4,210.00 (1,118.00, 15,3.7.75)	−5.786	< 0.001
Lac (mmol/L)	0.5–1.6	3.10 (2.35, 3.65)	2.15 (1.73, 3.25)	−1.959	0.050

NICCD, neonatal intrahepatic cholestasis caused by citrin deficiency; INC, idiopathic neonatal cholestasis; Mean ± SD denotes mean ± standard deviation; M (Q_1_, Q_3_) means median (the first quartile, the third quartile); HGB, hemoglobin; ALT, alanine aminotransferase; AST, aspartate aminotransferase; TB, total bilirubin; DB, direct bilirubin; IB, indirect bilirubin; TBA, total bile acid; GGT, gamma-glutamyl transpeptidase; AKP, alkaline phosphatase; ALB, albumin; PT, prothrombin time; APTT, activated partial thromboplastin time; FIB, fibrinogen; TT, thrombin time; INR, international normalized ratio; Glu, blood glucose; TCH, total cholesterol; TG, triglyceride; AMON, blood ammonia; AFP, alpha-fetoprotein; Lac, lactic acid.

### 3.4 SLC25A13 variants in the NICCD subjects

Due to constraints related to testing duration and costs, 8 patients underwent only Sanger sequencing. One patient, initially negative by Sanger sequencing, was subsequently subjected to multiplex PCR, which identified a homozygous positive result for the C.1751-5_1751-4insGATTTCTCCA insertion variant. The remaining 46 patients underwent NGS testing. Among the total cohort of 55 patients, 33 were evaluated for the C.1751-5_1751-4insGATTTCTCCA insertion variant, yielding results of 8 negative, 9 homozygous positive, and 16 heterozygous positive cases.

A total of 19 variant types of *SLC25A13* gene were identified in 55 NICCD patients (Table 3), which were inherited from at least one of their parents. These variations appeared a total of 88 times in all NICCD patients. The most common variant types were c.852_855del and C.1751-5_1751-4insGATTTCTCCA, accounting for 37.50% and 29.55%, respectively. There were 7 novel *SLC25A13* variant types. Among the novel variants, c.1196T>A, and c.15 + 1G>T were pathogenic variants. The c.475C>T, c.1649A>G, and c.761T>C were likely pathogenic variants. The variants c.1913T>G and c.848 + 6T>C were of uncertain pathogenicity.

**Table 3:** Summary of *SLC25A13* genetic variant types of the NICCD subjects.

No.	Variant type	Amino acid variation	Number of variants (*N*)	Percentage (*N*/88 total variant numbers)
1	c.852_855del (12)	p.Met285Profs × 2	33	37.50%
2	c.1751-5_1751-4insGATTTCTCCA (18)	Unknown	26	29.55%
3	c.615 + 5G>A (14)	Unknown	5	5.68%
4	c.1638_1660dup (12)	p.Ala554Glyfs × 17	5	5.68%
5	**c.475C**>**T**	p.Gln159Ter	3	3.41%
6	c.754G>A (19)	p.Glu252Lys	2	2.27%
7	c.550C>T (14)	p.Arg184Ter	2	2.27%
8	c.1051G>A (20)	p.Asp351Asn	1	1.14%
9	c.1402C>T (20)	p.Arg468Ter	1	1.14%
10	c.1778A>C (21)	p.Gln593Pro	1	1.14%
11	c.958C>T (7)	p.Arg320Ter	1	1.14%
12	c.1095del (22)	p.Phe365fs	1	1.14%
13	**c.1649A**>**G**	p.Gln550Arg	1	1.14%
14	**c.761T**>**C**	p. Phe254Ser	1	1.14%
15	**c.1913T**>**G**	p.Val638Gly	1	1.14%
16	Homozygous insertion variant of the long fragment in exon 5 and intron 5	Unknown	1	1.14%
17	**c.1196T**>**A**	p.Leu399Ter	1	1.14%
18	**c.15** **+** **1G**>**T**	Unknown	1	1.14%
19	**c.848** **+** **6T**>**C**	Unknown	1	1.14%

The detailed gene sequence of the 16th variant type could not be detected due to technical limitations. The novel 7 variants found in this study were indicated by bold letters. References were provided for the previously reported variants. The Genbank accession number of reference transcript for *SLC25A13* was NM_001160210.

### 3.5 Treatment outcome of the NICCD subjects

The biochemical changes and nutritional status of NICCD patients at 1, 3, and 12 months post-treatment were shown in Table 4. Compared with the baseline levels, the weight, WAZ, ALB, FIB, Glu, VA, VD and VE levels of NICCD patients at 1 month post-treatment were significantly increased, while the levels of AST, TB, DB, IB, TBA, GGT, AKP, PT, APTT, TT, TCH and AFP were significantly decreased (all *P* < 0.05). Ten indices including TB, IB, ALB, PT, APTT, FIB, TT, Glu, VA and VD were in their normal ranges at 1 month post-treatment.

**Table 4:** Biochemical changes and nutritional status of NICCD infants at 1-, 3-, and 12- months post treatment.

Index [reference range]	Before treatment	1-month post treatment [Mean ± SD, or M (Q1, Q3)]	*t/z* test	*P* value^a^	3-months post treatment [Mean ± SD, or M (Q1, Q3)]	*t/z* test	*P* value^b^	12-months post treatment [Mean ± SD, or M (Q1, Q3)]	*t/z* test	*P* value^c^	Normal control [Mean ± SD, or M (Q1, Q3)]	*t/z* test	*P* value^d^
Weight (kg)	4.40 (4.00, 5.20)	5.80 (5.20, 6.50)	−5.765	< 0.001	7.50 (6.90, 8.00)	−5.462	< 0.001	11.00 (10.00, 12.38)	−6.536	< 0.001	10.80 (9.93, 11.88)	−0.425	0.671
WAZ	−1.54 ± 0.82	−0.64 ± 0.64	−5.502	< 0.001	−0.03 ± 0.77	−3.580	0.001	0.07 ± 0.81	−0.531	0.597	−0.07 ± 0.47	0.740	0.463
ALT (U/L) [7–30]	43.00 (29.75, 57.25)	45.00 (25.00, 81.00)	−0.223	0.824	50.00 (31.00, 69.00)	−0.247	0.805	21.50 (18.25, 30.75)	−4.439	< 0.001	23.00 (15.00, 27.00)	−0.555	0.579
AST (U/L) [14–44]	116.00 (85.25, 135.75)	68.00 (54.00, 104.00)	−3.935	< 0.001	56.00 (44.00, 69.00)	−2.456	0.014	40.50 (37.25, 48.75)	−3.793	< 0.001	42.50 (38.25, 52.50)	−0.564	0.573
TB (μmol/L) [4.3–17.7]	151.40 (125.28, 197.70)	17.70 (9.10, 50.70)	−7.585	< 0.001	6.70 (5.50, 9.40)	−5.257	< 0.001	8.95 (6.77, 10.98)	−2.662	0.008	8.55 (6.95, 11.35)	−0.066	0.947
DB (μmol/L) [0–3.4]	71.70 (55.98, 97.45)	12.30 (5.60, 31.80)	−6.762	< 0.001	3.00 (2.60, 3.80)	−4.870	< 0.001	3.20 (2.43, 4.18)	−0.258	0.797	3.05 (2.08, 3.85)	−1.102	0.270
IB (μmol/L) [0–12]	78.10 (52.28, 105.03)	6.50 (3.70, 10.30)	−7.400	< 0.001	3.80 (2.30, 5.10)	−3.448	0.001	5.35 (4.30, 7.33)	−3.654	< 0.001	5.70 (4.23, 8.40)	−0.422	0.673
TBA (μmol/L) [0–10]	255.15 (197.15, 303.03)	43.00 (17.90, 164.70)	−5.846	< 0.001	6.40 (4.80, 11.30)	−5.110	< 0.001	4.45 (1.63, 6.35)	−3.271	0.001	3.40 (1.15, 6.33)	−0.704	0.481
GGT (U/L) [5–19]	188.50 (131.00, 231.00)	122.00 (59.00, 181.00)	−3.347	0.001	42.00 (28.00, 86.00)	−3.618	< 0.001	15.00 (14.00, 22.00)	−5.397	< 0.001	10.50 (9.00, 12.00)	−4.272	< 0.001
AKP (U/L) [143–406]	907.00 (723.50, 1,327.50)	409.00 (328.00, 482.00)	−6.953	< 0.001	339.00 (232.00, 390.00)	−2.919	0.004	249.50 (221.00, 331.50)	−1.8891	0.059	241.50 (225.00, 268.00)	−0.687	0.492
ALB (g/L) [39–54]	32.05 ± 4.37	42.33 ± 3.74	−11.458	< 0.001	44.31 ± 2.92	−2.470	0.016	46.39 ± 3.58	−2.607	0.011	45.18 ± 2.81	1.370	0.176
PT (s) [11.0–14.5]	15.70 (12.75, 18.55)	12.50 (11.50, 13.00)	−4.715	< 0.001	12.00 (11.50, 12.40)	−1.564	0.118	11.80 (11.45, 12.23)	−0.856	0.392	–	–	–
APTT (s) [28.0–44.5]	43.30 (36.80, 53.65)	37.50 (31.40, 40.30)	−4.047	< 0.001	34.35 (30.33, 39.90)	−0.721	0.471	32.35 (29.15, 36.68)	−1.253	0.210	–	–	–
FIB (g/L) [2–4]	1.09 (0.87, 1.38)	2.35 (1.99, 2.81)	−7.134	< 0.001	2.29 (1.81, 2.48)	−1.519	0.129	2.37 (2.20, 2.98)	−1.767	0.077	–	–	–
TT (s) [14–21]	19.60 (21.40, 23.40)	18.50 (16.90, 19.70)	−5.447	< 0.001	18.30 (17.40, 18.78)	−0.255	0.799	17.50 (16.50, 18.60)	−1.905	0.057	–	–	–
Glu (mmol/L) [3.9–5.8]	2.80 (1.90, 3.60)	4.10 (3.72, 4.70)	−4.559	< 0.001	4.70 (4.10, 5.10)	−2.277	0.023	4.70 (4.10, 5.15)	−0.070	0.944	–	–	–
TCH (mmol/L) [3.12–5.20]	4.35 (3.72, 5.62)	3.59 (2.93, 3.81)	−3.659	< 0.001	3.38 (2.88, 3.83)	−0.853	0.394	4.30 (3.82, 4.87)	−4.579	< 0.001	–	–	–
TG (mmol/L) [0.8–1.8]	0.92 (0.68, 2.37)	1.51 (1.02, 1.93)	−1.195	0.232	1.35 (0.94, 2.17)	−0.053	0.957	0.91 (0.70, 1.29)	−2.628	0.009	–	–	–
AFP (ng/ml) [0–12]	60,500.00 (33,737.25, 60,500.00)	3,102.50 (463.75, 15,062.25)	−5.700	< 0.001	52.29 (29.19, 142.20)	−5.472	< 0.001	4.42 (1.69, 7.10)	−5.389	< 0.001	–	–	–
VA (μmol/L) [1.05–2.80]	0.58 (0.43, 0.80)	1.82 (1.43, 2.33)	−5.406	< 0.001	2.32 (1.98, 2.77)	−2.048	0.041	2.80 (2.06, 3.16)	−1.976	0.048	2.28 (2.02, 2.60)	−2.073	0.038
VD (nmol/L) [75–250]	54.23 (43.49, 92.15)	96.87 (73.67, 141.05)	−3.345	0.001	117.45 (101.93, 151.50)	−1.607	0.108	139.05 (107.98, 173.68)	−1.297	0.195	121.60 (107.58, 151.73)	−1.158	0.247
VE (μmol/L) [16.25–33.50]	17.72 (15.63, 21.25)	35.17 (21.73, 44.28)	−3.956	< 0.001	33.16 (25.85, 40.38)	−0.258	0.796	30.39 (24.68, 36.63)	−1.051	0.293	28.84 (25.69, 31.36)	−0.772	0.440
Pb (μg/L)	–	–	–	–	–	–	–	29.79 ± 12.09	–	–	28.10 ± 10.22	0.512	0.611
Zn (μmol/L)	–	–	–	–	––	–	–	4.15 ± 0.77	–	–	4.00 ± 0.92	0.592	0.557
Fe (μmol/L)	–	–	–	–	–	–	–	417.12 ± 27.47	–	–	425.27 ± 23.54	−1.085	0.284
Cu (μmol/L)	–	–	–	–	–	–	–	1.06 ± 0.13	–	–	1.07 ± 0.12	−0.306	0.761

WAZ, weight for age z-score; ALT, alanine aminotransferase; AST, aspartate aminotransferase; TB, total bilirubin; DB, direct bilirubin; IB, indirect bilirubin; TBA, total bile acid; GGT, gamma-glutamyl transpeptidase; AKP, alkaline phosphatase; ALB, albumin; PT, prothrombin time; APTT, activated partial thromboplastin time; FIB, fibrinogen; TT, thrombin time; Glu, blood glucose; TCH, total cholesterol; TG, triglyceride; AFP, alpha-fetoprotein; VA, vitamin A; VD, vitamin D; VE, vitamin E; Pb, Zn, Fe and Cu means lead, zinc, iron, and copper, respectively.

^a^ Represents the comparison between before treatment and 1 month after treatment.

^b^ Represents the comparison between 1 month after treatment and 3 months after treatment.

^c^ Represents the comparison between 3 months and 12 months after treatment.

^d^ Represents the comparison between 12 months after treatment and normal control. “-” indicates no data. NICCD, neonatal intrahepatic cholestasis caused by citrin deficiency. Mean ± SD denotes mean ± standard deviation. M (Q_1_, Q_3_) means median (the first quartile, the third quartile).

The weight, WAZ, ALB, Glu and VA levels of NICCD patients were significantly increased at 3 months post-treatment compared with those at 1 month post-treatment (all *P* < 0.05). In addition, the levels of AST, TB, DB, IB, TBA, GGT, AKP and AFP at 3 months post-treatment showed significant decrease compared with those at 1 month post-treatment (all *P* < 0.05). Three indices (i.e., DB, TBA and AKP) were further changed to their normal ranges at 3 months after treatment.

The weight was significantly increased, and ALT, AST, GGT and AFP levels were significantly decreased to their normal ranges at 12 months post-treatment compared with those at 3 months post-treatment (all *P* < 0.05). The levels of TCH, TG, and VE remained within the normal range before and throughout the treatment. Besides, NICCD patients showed comparable levels of Pb, Zn, Fe, and Cu compared to normal controls (*P* > 0.05). Overall, all biochemical and nutritional indices were within normal levels at 12 months post-treatment. No significant differences were found between NICCD patients treated for 12 months and normal controls, except for GGT and VA levels, indicating a good treatment outcome in NICCD patients.

### 3.6 Long term follow-up results of the NICCD subjects

All NICCD patients were followed up until death or at least 1 year of age, with the longest follow-up of 4 years old. Two cases carrying c.852_855del/c.1196T>A and c.754G>A/C.1751-5_1751-4insGATTTCTCCA showed growth retardation in early childhood and developed FTTDCD. Eight cases had special dietary habits, and they preferred high-protein and low-carbohydrate diets. Their SLC25A13 genetic variant types were c.15 + 1G>T/c.1402C>T, C.1751-5_1751-4insGATTTCTCCA, c.852_855del, c.852_855del/c.958C>T, c.852_855del/C.1751-5_1751-4insGATTTCTCCA, c.1649A>G/C.1751-5_1751-4insGATTTCTCCA, and c.852_855del/c.1913T>G. One case died from cirrhosis, and he showed c.852_855del/C.1751-5_1751-4insGATTTCTCCA compound heterozygous variants. No adverse events were found in other patients.

## 4 Discussion

Cholestasis is a common cause of liver diseases in children, with a morbidity of approximately 1/2,500 (23). Great differences exist in the treatment and prognosis of cholestasis with different etiologies. Early identification of the etiologies and targeted treatment are of great significance in improving the prognosis of cholestasis in children. NICCD is one of the principal etiologies of intrahepatic cholestasis in infancy (24). However, the infants with NICCD manifest similar clinical symptoms as INC infants, therefore, differential diagnosis is still needed. Although differences in some indices between NICCD and INC patients have been previously reported (4, 25, 26), the small sample size of those studies precluded a definite conclusion. In this study, the patient cohorts of NICCD and INC were large enough to test the previous findings.

The comparisons of epidemiological characteristics, nutritional status and biochemical indices in the NICCD and INC infants were performed in the present study. Patients in the two groups showed similar epidemiological characteristics including gender, onset age, and age at presentation. Studies have shown that the birth weight and length of NICCD infants were lower than that of healthy newborns at the same gestational age, showing intrauterine growth retardation (27, 28). Consistently, in our study, NICCD patients showed significantly decreased birth weight and HAZ compared to healthy peers. The malnourished and stunted patients in NICCD group were respectively 1.3-fold and 1.8-fold higher than that in INC group. Citrin deficiency has been reported to limit fetal growth in early pregnancy because mitochondria could not produce enough energy for infants (27). In patients with cholestasis, the obstruction of bile discharge affects the absorption of fat, which weakens the absorption of fat-soluble vitamins (29). In this study, patients in both the NICCD and INC groups showed varying degrees of decrease in VA, VD and VE levels. Among them, the cases with decreased VA levels in NICCD group was 1.9-fold higher than that in INC group (89.6% vs. 47.4%). These findings indicated that attention should be paid to infants with malnutrition, growth retardation and severely decreased VA levels.

Some biochemical indices in this study were significantly different among NICCD and INC groups, which may be meaningful for the diagnosis of NICCD. A previous study with a small number of subjects showed that NICCD patients had lower ALT and AST levels, as well as a higher AST to ALT ratio compared with INC patients (24). Wang et al. also found that the ALT and AST levels were increased in both NICCD and INC groups, with a higher level in INC group compared to NICCD group (30). The results in our study were consistent with previous findings. It has been reported that higher AST levels than ALT levels reflect more severe liver cell damage (31). Bilirubin and TBA were the major elevated indicators of cholestasis (32). Significantly higher TB, IB, and TBA levels were found in NICCD patients compared to INC patients, suggesting that the disorders in bilirubin conversion and bile acid excretion in the NICCD group were more severe than those in the INC group. Besides, ALB and Glu in NICCD group was significantly lower than that in INC group, which were consistent with the previous description that low ALB and Glu levels were also associated with NICCD (33, 34). Additionally, compared with INC group, PT and TT were significantly increased in NICCD group, together with significant decrease of FIB. These may be related to the fact that citrin deficiency resulted in insufficient aspartate in the cellular cytoplasm, leading to reduced protein synthesis, hypoalbuminemia and coagulation abnormalities. Besides, citrin protein provides substrate for gluconeogenesis in the pathway for the conversion of amino acids to glucose. Its deficiency can cause disorders in gluconeogenesis, resulting in hypoglycemia. Taken together, NICCD patients showed more severe impairments in bile acid excretion, bilirubin metabolism, liver protein synthesis, glucose metabolism, and coagulation function than INC patients. This may be related to disorders in cellular metabolism and oxidation/reduction homeostasis caused by citrin deficiency (35, 36). It was worth mentioning that sufficient attention should be paid in AFP among the series of biochemical indices. The median AFP concentration was 60,500 ng/ml in NICCD patients but was only 4,210 ng/ml in INC patients. Therefore, AFP could be used as one of the considerable indicators for distinguishing NICCD from INC.

It is indeed difficult for pediatricians to make an accurate diagnosis of NICCD based just on clinical manifestations and laboratory changes. The definitive diagnosis of this inherited disease relies on the variant analysis of the causative gene *SLC25A13*. We confirmed the diagnosis of NICCD by *SLC25A13* variant analysis in this study. Among the 19 variant types, c.852_855del and C.1751-5_1751-4insGATTTCTCCA were the most common variants, which were consistent with the previous studies (21, 32). In addition, we found 7 novel variant types that have never been reported previously, including c.475C>T, c.1196T>A, c.15 + 1G>T, c.1649A>G, c.761T>C, c.1913T>G, and c.848 + 6T>C. These new findings enriched the variant spectrum of the *SLC25A13* gene and provided a reference for the definite diagnosis of NICCD, which can also be a valuable method for the epidemiological investigation of citrin deficiency in special populations.

There are currently no specific treatment options for most inherited metabolic liver diseases. NICCD patients were usually treated with a dietary management of lactose-free and MCT-enriched formula (3, 37). The majority of NICCD patients in our study experienced no adverse events 12 months post-treatment, supporting the effectiveness of this dietary management approach. Previous studies reported that a small number of children suffered from severe liver failure or infection-related death due to late diagnosis and untimely dietary management (38–40). Unfortunately, one NICCD case in this study died from cirrhosis at less than one month after treatment. He had the latest age at presentation (1 year and 4 months) and the highest AST (277 U/L), TB (427.3 μmol/L) and DB (316.2 μmol/L) levels at diagnosis among all NICCD patients. This may be related to the prolonged cholestasis and repeated episodes of steatohepatitis resulting from delayed diagnosis and treatment. Besides, the development of FTTDCD in two NICCD patients raises important considerations regarding the underlying genetic factors. Both patients harbor heterozygous variants, including c.852_855del/c.1196T>A and c.754G>A/C.1751-5_1751-4insGATTTCTCCA. These variants may significantly disrupt the function of the *SLC25A13* gene. The c.852_855del frameshift variant leads to a premature stop codon, likely resulting in a truncated or nonfunctional protein, severely compromising citrin's role in hepatocyte metabolism. Similarly, the c.1196T>A missense variant could alter amino acid residues critically for proper protein conformation and activity. These alterations may exacerbate metabolic imbalances in NICCD patients, contributing to the progression toward FTTDCD due to impaired energy metabolism and ammonia detoxification. In the second case, the c.754G>A missense variant, in conjunction with the intronic insertion C.1751-5_1751-4insGATTTCTCCA, might lead to aberrant splicing, reducing the production of functional citrin. The decreased mitochondrial aspartate transport would impair the urea cycle and gluconeogenesis, worsening metabolic derangements. This metabolic disruption likely plays a role in the development of growth retardation and hepatic dysfunction seen in FTTDCD. Eight cases preferred high-protein and low-carbohydrate diets. This special dietary preference may be related to the fact that NICCD children can supplement aspartic acid and arginine after ingesting such foods, which is beneficial to promote urea cycle, reduce blood ammonia, and relieve symptoms (20). Overall, NICCD children should be diagnosed in time and dietary management should be given to avoid exacerbation of the disease.

Despite our efforts to analyze the outcomes of NICCD patients, there remain certain limitations in our study. One of the primary challenges is the limited availability of detailed patient information, which restricts the depth of analysis regarding specific outcomes, such as survival rates and contributing factors to varying prognoses. As a result, while our findings offer initial insights, further research is necessary to explore additional clinical data, which may provide a more comprehensive understanding of NICCD and its long-term outcomes. Future studies with larger cohorts and more detailed patient follow-up will be essential to overcoming these limitations.

## 5 Conclusion

In summary, NICCD infants showed more severe impairments in liver, coagulation, and metabolic function than INC. Malnutrition, growth retardation, severely decreased VA levels, and significantly increased AFP levels could provide reference for the diagnosis and differential diagnosis of NICCD. *SLC25A13* variant analysis should be considered as a reliable tool for definitive diagnosis. More attention should be paid to c.1196T>A and c.15 + 1G>T among the 7 novel variants as they were related to FTTDCD and special dietary habits. Lactose-free and MCT-supplemented formula could improve energy metabolism of the liver and was recommended for NICCD patients.

## Data Availability

The original contributions presented in the study are included in the article/Supplementary Material, further inquiries can be directed to the corresponding author/s.
